# Epigenetic and metabolic plasticity of tumor cells in immunotherapy resistance

**DOI:** 10.3389/fimmu.2026.1968997

**Published:** 2026-10-05

**Authors:** Jee-Eun Choi, Inwha Baek

**Affiliations:** 1 Department of Pathology, School of Clinical Medicine, The University of Hong Kong, Queen Mary Hospital, Hong Kong, Hong Kong SAR, China; 2 Centre for Oncology and Immunology, Hong Kong Science Park, Hong Kong, Hong Kong SAR, China; 3 College of Pharmacy, Kyung Hee University, Seoul, Republic of Korea; 4 Institute of Regulatory Innovation through Science (IRIS), Kyung Hee University, Seoul, Republic of Korea; 5 Regulatory Science, Department of Pharmacy, Graduate School, Kyung Hee University, Seoul, Republic of Korea

**Keywords:** cancer immunotherapy, chimeric antigen receptor-T cell therapy, drug resistance, epigenetic plasticity, immune checkpoint blockade, metabolic plasticity

## Abstract

Cancer immunotherapy has demonstrated remarkable efficacy across multiple tumor types. Despite their significant clinical benefits, most patients with solid tumors remain refractory to immunotherapy, and initial responders frequently develop acquired resistance. Tumor plasticity is a key driver of resistance to cancer immunotherapy. In this review, we discuss how epigenetic and metabolic plasticity of tumor contributes to immunotherapy resistance and highlight emerging strategies targeting these programs to overcome resistance and improve clinical outcomes.

## Introduction

Cancer immunotherapy, including immune checkpoint blockade (ICB) and chimeric antigen receptor (CAR)-T cell therapy, has established a new paradigm for cancer therapy. ICB targeting programmed cell death protein 1 (PD-1), its ligand PD-L1, or cytotoxic T-lymphocyte-associated protein 4 (CTLA-4) has produced durable clinical responses across diverse cancer types (1–7). CAR-T therapy has demonstrated unprecedented efficacy in hematological malignancies, particularly in patients with refractory B-cell leukemias and lymphomas (8–10). Despite these remarkable clinical successes, many patients are refractory or eventually develop acquired resistance to immunotherapy (11, 12). Moreover, the clinical benefit of immunotherapy remains limited in most solid tumors.

Resistance to immunotherapy can arise from both tumor-intrinsic and tumor-extrinsic mechanisms that allow cancer cells to escape immune surveillance. In this review, we focus on tumor-intrinsic plasticity that confers immunotherapy resistance (13). These adaptations include downregulation of antigen processing and presentation, disruption of interferon signaling, reduced production of signals required for immune cell recruitment and infiltration, and resistance to T cell-mediated cytotoxicity. Importantly, these tumor-intrinsic adaptations can be driven by epigenetic and metabolic rewiring and their crosstalk (14), which regulate immune evasion (15–20). Epigenetic remodeling reshapes the transcriptional landscape governing immunologic recognition and responsiveness, while metabolic rewiring alters nutrient availability and metabolite accumulation within the tumor immune microenvironment (TIME), thereby modulating antigen presentation and effector T cell function. In this review, we discuss how tumor-intrinsic epigenetic and metabolic reprogramming promotes resistance to immunotherapy and highlight emerging therapeutic strategies that target tumor cell plasticity to enhance the efficacy of cancer immunotherapy.

## Epigenetic plasticity in cancer affects immunotherapy resistance

Tumor cells dynamically remodel their interactions with immune cells within the TIME to evade immune surveillance and acquire resistance to cancer immunotherapies (11, 21, 22). One of the key mechanisms underlying this immunomodulatory capacity is epigenetic reprogramming, whereby DNA methylation, histone modifications, and chromatin remodeling reshape transcriptional programs in cancer cells, ultimately affecting the cellular states and functions of surrounding immune cells (23, 24) (**Figure 1**). Such examples include suppression of immunogenic antigen expression, downregulation of antigen-presenting machinery, and reduced chemokine secretion that recruits T cells (**Figure 1**). This limits the recruitment and activation of cytotoxic T cells and fosters an immunosuppressive TIME.

**Figure 1:**
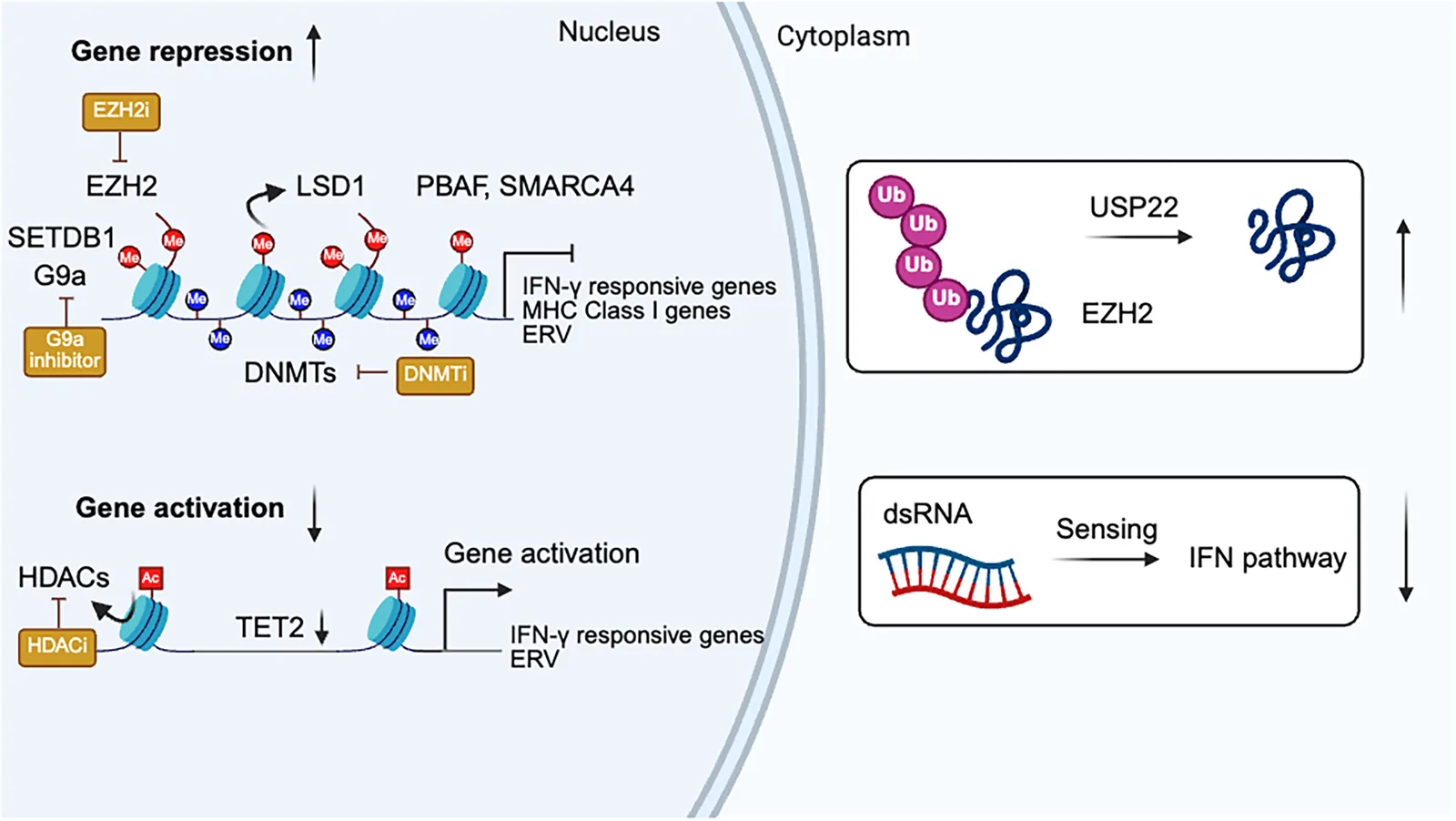
Epigenetic reprogramming of tumor cells contributes to immunotherapy resistance. Alterations in DNA methylation, histone modifications, and chromatin remodeling in tumor cells downregulate the expression of IFN- γ responsive genes, MHC class I genes, and ERVs leading to resistance to cancer immunotherapy. The histone methyltransferases EZH2, SETDB1, and G9a and the histone demethylase LSD1 promote transcriptional repression, whereas the PBAF chromatin-remodeling complex containing SMARCA4 regulates chromatin states. DNA methyltransferases further induce transcriptional repression. In contrast, histone deacetylases and reduced activity of TET2 attenuate transcription activation of IFN- γ responsive genes and ERVs. In the cytoplasm, USP22-mediated deubiquitination stabilizes EZH2, promoting its accumulation, whereas decreased sensing of ERV-derived dsRNA reduces the IFN response. Pharmacological inhibition of EZH2, G9a, DNMTs, or HDACs can reverse these epigenetic programs and restore tumor immunogenicity. Upward and downward arrows indicate increased and decreased responses, respectively. (EZH2, enhancer of zeste homolog 2; PBAF, polybromo and BRG1-associated factor; IFN- γ, interferon-gamma; MHC, major histocompatibility complex; ERV, endogenous retrovirus; Ub, ubiquitin; dsRNA, double-stranded RNA; DNMTi, DNA methyltransferase inhibitor; EZH2i, EZH2 inhibitor; HDACi, histone deacetylase inhibitor).

Genome-wide CRISPR-Cas9 screens have identified several epigenetic regulators that govern T cell-dependent anti-tumor response and resistance to immune checkpoint blockade (ICB) (25–27). Among the major resistance factors is the polybromo and BRG1-associated factors (PBAF) complex, a subtype of the SWI/SNF chromatin remodeling complex. In the murine B16F10 melanoma cell line, inactivation of PBAF components, including *Arid*2 and *Pbrm1*, upregulated the expression of interferon (IFN)-γ-responsive genes, such as *Cxcl9* and *Cxcl10*, which encode key chemokines that recruit effector T cells into the TIME (25). Disruption of the PBAF complex increased T cell infiltration and potentiated anti-tumor immune responses (25). Alterations in SWI/SNF chromatin remodeling complexes that are associated with the immune-active tumor microenvironment further underscore the connection between chromatin regulation and ICB resistance. *SMARCA4*-mutated tumors are characterized by increased T cell infiltration and enhanced clinical responsiveness to ICB (28). In murine ovarian cancer models, *Smarca4* deficiency activates the RNA-sensing pathway, leading to enhanced expression of type I IFN-stimulated genes and major histocompatibility complex (MHC) class I, thereby increasing tumor immunogenicity *in vivo* (29). Similarly, other SWI/SNF subunits, including ARID1A, are negatively associated with type I IFN production in tumor cells (30). These findings identify SWI/SNF complexes as important regulators of tumor immunogenicity and determinants of resistance to immunotherapy.

Another CRISPR-Cas9 screen targeting epigenetic regulators associated with ICB resistance identified SETDB1, a histone H3-lysine 9 (H3K9) methyltransferase and the catalytic subunit of the KAP1 and HUSH complexes, as a key mediator of tumor immune evasion (31). SETDB1 suppresses the expression of immunogenic transposable elements (TEs) that mimic viruses and upregulate type I IFN responses. Consequently, the generation and presentation of TE-derived antigens on MHC class I molecules are reduced, impairing tumor recognition by CD8^+^ T cells and attenuating anti-tumor immune responses. Mechanistically, SETDB1 is recruited to TE loci by KDM5B, a histone H3-lysine 4 (H3K4) demethylase in a demethylase-independent manner (32). Notably, KDM5B expression is inversely correlated with clinical responsiveness to ICB in patients with melanoma. A loss of KDM5B induces a robust type I IFN response in the melanoma model. These findings establish the KDM5B-SETDB1 axis as a critical epigenetic regulator of tumor immune evasion and immunotherapy resistance.

Reduced MHC class I expression represents a major mechanism of resistance to ICB by limiting neoantigen presentation and subsequent activation of cytotoxic CD8^+^ T cells. Multiple epigenetic regulators contribute to intratumoral transcriptional silencing of MHC class I genes. For example, enhancer of zeste homolog 2 (EZH2), the catalytic subunit of Polycomb repressive complex 2 (PRC2), represses MHC class I expression through histone H3-lysine 27 (H3K27) trimethylation and chromatin compaction (17, 33). Ubiquitin-specific peptidase 22 (USP22), a deubiquitinating enzyme is also associated with ICB resistance. USP22 enhances EZH2-mediated downregulation of MHC class I expression and reduces intratumoral CD8^+^ T cell infiltration by deubiquitinating and protecting EZH2 from proteasomal degradation (34). Usp22 inhibition combined with anti-PD-1 therapy exhibits greater tumor regression than anti-PD-1 therapy alone (34).

An increasing body of work suggests that other posttranslational modifications (PTMs), including SUMOylation and lactylation, also contribute to immune evasion and resistance to immunotherapy (35–39). Increased SUMOylation in cancer cells promotes immune evasion by suppressing the MHC class I antigen presentation (36). In hepatocellular carcinoma (HCC) cells, H3K18 lactylation increases chromatin accessibility at the promoters of transcriptional repressor GATA binding 1 (TRPS1) and E-twenty-six transformation-specific variant 1 (ETV1), leading to their upregulation (40). TRPS1 confers resistance to CD8^+^ T cell-mediated cytotoxicity through a GLUT1-dependent glycolytic positive feedback loop, whereas ETV1 inhibits CD8^+^ T cell infiltration by enhancing chemokine (C-C motif) ligand 2 (CCL2) transcription. A GLUT1 inhibitor and anti-CCL2 antibody combined with anti-PD-1 therapy enhances antitumor immunity and improves ICB response. Another study using human HCC cells demonstrated that H3K18 lactylation increases the expression of major vault protein, which suppresses CD8^+^ T cell-mediated antitumor activity by stabilizing PD-L1 (41). Given the emerging roles of SUMOylation and lactylation in tumor-intrinsic immune evasion, the potential contribution of these PTMs to immunotherapy resistance is an intriguing topic for further investigation.

DNA methylation dynamics also play an essential role in regulating tumor immunogenicity. Reduced activity of ten-eleven translocation methylcytosine dioxygenase 2 (TET2) attenuates IFN-γ-JAK-STAT signaling in tumor cells, resulting in decreased expression of T helper 1 (Th1)-associated chemokines and reduced infiltration of tumor-infiltrating lymphocytes (TILs) (42). Deletion of Tet2 in murine melanoma and colon cancer models confers resistance to anti-PD-L1 therapy, highlighting the importance of active DNA demethylation in maintaining anti-tumor immune responses (42). Taken together, accumulating evidence demonstrates that epigenetic regulators are associated with ICB resistance through multiple mechanisms of immune evasion.

## Epigenetic drugs synergize with cancer immunotherapy

Growing evidence indicates that epigenetic reprogramming is a central driver of resistance to cancer immunotherapy, providing a compelling rationale for combining epigenetic therapies (epidrugs) with ICB to restore tumor immunogenicity and improve therapeutic efficacy. Among the most extensively investigated combinations are DNA methyltransferase (DNMT) inhibitors. In patients with relapsed or refractory NK/T-cell lymphoma, DNMT inhibition combined with anti-PD-1 immunotherapy enhances the expression of endogenous retroviral elements (ERVs), thereby activating viral mimicry and type I IFN signaling, which in turn increases tumor sensitivity to PD-1 blockade (15, 43). Likewise, combined inhibition of DNMTs and histone deacetylases (HDACs) activates ERV transcription and type I IFN signaling in non-small cell lung cancer (NSCLC) cells (44). This transcriptional activation leads to increased expression of the T cell chemoattractant CCL5 and enhanced recruitment of CD8^+^ TILs into the tumor microenvironment. Additionally, combined epidrugs suppress the expression of exhaustion-associated genes while promoting the expression of activation- and memory-associated genes, thereby improving the durability of responses to ICB.

HDAC inhibitors alone also remodel the TIME to potentiate immunotherapy. In murine SM1 melanoma, the selective HDAC6 inhibitor Nexturastat A combined with anti-PD-1 blockade significantly increased the infiltration of CD8^+^ T cells and natural killer (NK) cells while simultaneously neutralizing increased expression of PD-L1 and MHC class I induced by anti-PD-1 blockade (45). These changes are accompanied by a reduction in pro-tumoral M2 macrophages, which are key immune cells involved in immune evasion and tumor progression. Tumor-intrinsic regulation of macrophage recruitment is another mechanism of immune evasion. For example, overexpression of FBXL16 in glioma cell lines inhibits cytokine release, thereby limiting the recruitment of M2 macrophages to the TIME and reducing immune evasion (46).

Several studies have further demonstrated that targeting histone methylation enhances responsiveness to ICB. EZH2 inhibition significantly restores MHC class I expression in human and murine head and neck squamous cell carcinoma (HNSCC) cells, which leads to increased antigen-specific CD8^+^ T cell proliferation and IFN-γ production (47). Combined treatment with an EZH2 inhibitor and anti-PD-1 therapy suppresses tumor growth in anti-PD-1-resistant HNSCC models (47). Similarly, the EZH2 inhibitor GSK126 sensitizes NSCLC to PD-1 blockade by inducing double-strand RNA (dsRNA)-IFN pathway stress and upregulating antigen presentation gene expression (48, 49). Dual inhibition of EZH2 and the histone methyltransferase G9A using HKMTI-1–005 upregulates the expression of immunostimulatory genes, including the chemotactic chemokines (*Cxcl9*, *Cxcl10*, *Cxcl11*, and *Ccl5*) and ERV-K endogenous retroviral family transcripts in a murine ovarian cancer model (50). These transcriptional changes increase infiltration of cytotoxic T lymphocytes and NK cells while reducing tumor-promoting FoxP3^+^ CD4 T cells (50). In addition, depletion of the H3K4 demethylase LSD1 promotes the viral mimicry pathway and restores sensitivity to PD-1 blockade in refractory B16 melanoma tumors (16).

Collectively, epigenetic therapies enhance the efficacy of cancer immunotherapy through multiple complementary mechanisms, including restoration of viral mimicry and IFN signaling, reactivation of antigen presentation machinery, and induction of immunostimulatory chemokines. These transcriptional changes lead to extensive remodeling of the TIME. Combination therapy with epidrugs and ICB would serve as a promising approach to sensitizing tumor cells to ICB.

## Metabolic plasticity in cancer affects immunotherapy resistance

Metabolic reprogramming serves as one of the cancer hallmarks (21). A substantial body of literature supports that aberrant uptake or export of metabolites by cancer cells dynamically regulates nutrient availability within the TIME, where immune cells need to infiltrate, propagate, and be activated to exert immunologic functions against cancer cells (38) (**Figure 2**). In this section, relevant published works with *in vivo* preclinical validation are highlighted to emphasize translational potential of metabolic inhibitors in combination with immunotherapy.

**Figure 2:**
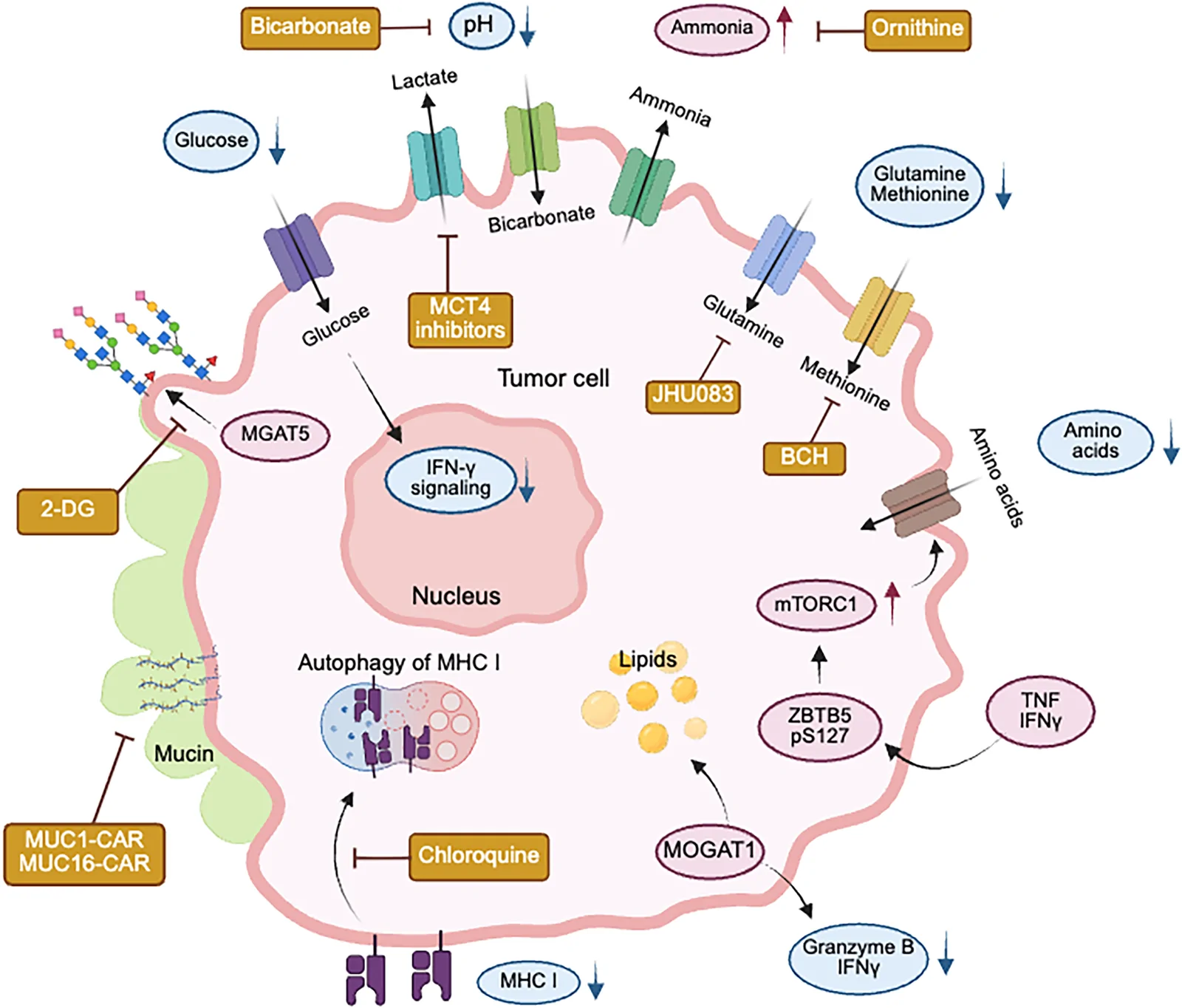
Metabolic reprogramming of tumor cells contributes to immunotherapy resistance. Tumor-driven aberrant uptake or secretion of metabolites regulates metabolite availability within the tumor immune microenvironment, affecting T cell survival, growth, and function. Decreased level of glucose in TIME can downregulate tumor-intrinsic IFN-γ pathway and anti-tumor immunity. Low pH in TIME through excess excretion of lactate or uptake of bicarbonate negatively impacts T cell-mediated cytotoxicity. Cancer cells with high amino acid metabolic activities aberrantly uptake glutamine or methionine and excrete ammonia, changing their levels in TIME. Moreover, tumor-intrinsic mTORC1 activation limits amino acid availability in TIME. Such metabolite availability in TIME inhibits T cell function and promotes immune evasion. T-cell-derived IFN-γ inhibits tumor’s ferroptosis defense system by downregulating cystine uptake. Lipid anabolic enzyme MOGAT1 restricts CD8^+^ T cell infiltration. MHC class I-mediated antigen presentation by tumor cells also affect T cell-mediated immunotherapy response through tumor-intrinsic autophagy machinery. *N*-linked glycans and *O*-linked glycans negatively impact CAR-T therapy response, which on the other hand, reveals a potential therapeutic targeting strategy. (2-DG, 2-deoxy-glucose; MGAT5, mannoside acetyl-glucosaminyltransferase 5; MHC, major histocompatibility complex; IFN- γ, interferon-gamma; MCT4, monocarboxylate transporter 4; MOGAT1, monoacylglycerol *O*-acyltransferase 1; TNF, tumor necrosis factor; BCH, 2-amino-2-norbornanecarboxylic acid).

Cancer cells often upregulate glucose metabolism, mainly through glycolysis, resulting in increased lactate production and excretion into the TIME. Avid glucose uptake in cancer cells and related metabolic competition within the TIME restrict effective anti-tumor immune responses by reducing tumor-infiltrating lymphocyte function (51). On the other hand, highly glycolytic cancer cells autonomously develop resistance to T cell-mediated cytotoxicity regardless of glucose availability within TIME, via downregulation of the IFN-γ signaling pathway (52). Lactate, one of the major metabolites secreted into TIME, and associated low pH within TIME also contribute to immunosuppression. Increased levels of lactate can inhibit T and NK cell survival and proliferation (53). An acidic pH driven by excess lactate secretion from tumor cells inhibits T cell function and infiltration, which can be mitigated by neutralizing acidity with bicarbonate therapy (54). Combination therapy using an lactate dehydrogenase (LDH) inhibitor or bicarbonate with immunotherapy can improve anti-tumor immune responses by overcoming resistance (52, 54). Tumor type-specific transporters responsible for the acidic TIME have been identified as targets to improve the efficacy of immunotherapy. In colorectal cancer, lactate efflux transporter monocarboxylate transporter 4 (MCT4) is a main transporter contributing to lactate efflux and acidic TIME (55). MCT4 blockade together with an immune checkpoint PD-L1 inhibitor attenuated tumor growth *in vivo* by increasing intratumoral pH and T cell infiltration/activation. In pancreatic cancer, bicarbonate influx transporter solute carrier family 4 member 4 (SLC4A4) in epithelial ductal cells can lead to acidification of TIME through active influx of bicarbonate into cancer cells (56). Inhibition of SLC4A4 in combination with immune checkpoint blockade can overcome immunotherapy resistance, resulting in increased survival *in vivo*.

Diverted from glycolysis, glycan production and proper glycosylation of cell surface proteins are important for antigen recognition by specific immunologic receptors as well as immune cell infiltration (57). Aberrant extracellular *N*-glycans mediated by mannoside acetyl-glucosaminyltransferase 5 (MGAT5) in pancreatic cancer cells confer resistance to CAR-engineered T cell-mediated anti-tumor responses (58). Targeting the *N*-glycans with a glycolysis inhibitor, 2-deoxy-*D*-glucose, in combination with different CAR-T therapies that are unique to different solid cancer types (e.g. CD44v6 and CEA) can overcome the resistance *in vivo* (58). MGAT5-dependent *N*-glycans protect tumor cells from pro-death stimuli, whereas deletion of *MGAT5* can re-sensitize immunotherapy-resistant pancreatic cancer cells to immune checkpoint blockade (59). These studies emphasize critical roles for *N*-glycans in mediating immunotherapy resistance, revealing an attractive therapeutic vulnerability in this type of immune-cold cancers. Cancer-associated mucins, heavily *O*-glycosylated proteins, can work as physical barriers for immune cell infiltration and antigen engagement by immune cells (60). To overcome low efficacy in immunotherapy, a series of CAR-T-based therapies co-targeting cancer-specific mucins (e.g. MUC1 and MUC16) and tumor-associated antigens has been developed (61–64). Some of these CAR-T therapeutics have undergone or are currently undergoing phase I/II clinical trials (NCT04025216, NCT05812326, NCT03525782, NCT06469281, and NCT07480954).

In addition to glucose, amino acids and their byproducts (e.g. ammonia) play key roles in modulating anti-tumor immune responses. Glutamine is one of the amino acids found at decreased levels in tumor interstitial fluid within the TIME in the immunocompetent murine model of colorectal cancer (65). Glutamine-addicted cancers, such as triple-negative breast cancer (TNBC), deplete glutamine within the TIME, thereby decreasing tumor-infiltrating T cell cytotoxicity (66). Glutamine blockade generates a more immune-reactive TIME by activating cytotoxic T cells while inhibiting tumor cell growth (67). Inhibition of glutamine metabolism provides an attractive metabolic checkpoint to overcome resistance to immunotherapy.

Ferroptosis, a unique cell death mechanism driven by iron-dependent lipid peroxidation (68), acts as an important determinant of therapeutic efficacy (69). IFN-γ from CD8^+^ T cells downregulates SLC3A2 and SLC7A11 expression in cancer cells, both of which serve as critical subunits of the cystine importer system that protects cancer cells from ferroptosis, thereby sensitizing cancer cells to ferroptosis during immunotherapy (70). As such, depletion of cystine or cysteine by cyst(e)inase (71) synergistically improves anti-tumor immunity in combination with anti-PD-L1 therapy through ferroptosis induction (70).

Dietary interventions could transiently modulate nutrient availability within TIME, thereby affecting cancer and immune cell behaviors. Chen et al. reported that a brief, 16 hours of fasting can promote the efficacy of immunotherapy by creating isoleucine-rich TIME in both mice and humans (72). Increased metabolic stress in cancer cells caused by short-term starvation releases isoleucine into TIME, through which CD8^+^ T cell-mediated cytotoxicity is enhanced. Importantly, this intervention also increases CD8^+^ T cell-mediated cytotoxic programs in patients with neoadjuvant immunotherapy. Exacerbated amino acid uptake in tumor cells through inflammatory cytokine-induced mTORC1 activity restricts amino acid availability within TIME, conferring immune evasion and resistance to immune checkpoint inhibitors (73). The molecular circuit (ZBTB5-pS127-mTORC1) responsible for the nutritional insufficiency in TIME exposes a therapeutic vulnerability to overcome cancer immunotherapy resistance (73). Ammonia is one of the byproducts from nitrogen-dependent metabolic processes, predominantly produced by amino acid metabolism. In advanced colorectal cancer, ammonia is accumulated by downregulation of HNF4A and subsequently the ammonia clearance pathway in cancer cells (74). Increased ammonia levels within TIME specifically induce growth retardation of CD4^+^ and CD8^+^ T cells as well as their exhaustion, while growth of colon cancer cells, neutrophils, and macrophages is minimally impacted (74). Detoxification of ammonia can restore sensitivity to immune checkpoint blockade with increased T cell infiltration and cytotoxic activity *in vivo*.

*In vivo* transcriptomic analyses in progressing breast cancer discovered monoacylglycerol *O*-acyltransferase 1 (MOGAT1), an enzyme involved in triglyceride synthesis, as a crucial mediator of immune evasion (75). MOGAT1-overexpressing cancer cells could not only promote neutral lipid anabolism and growth but also inhibit CD8^+^ T cell infiltration and T cell-mediated cytotoxicity. Inhibition of MOGAT1 together with PD-1 blockade restores immunotherapy sensitivity through an increased pool of highly activated T cells in TIME.

Inactivation of the MHC class I antigen presentation pathway is a well-established resistance mechanism to immunotherapy through loss-of-function mutations or transcriptional downregulation (76–79). Promoting degradation of MHC class I molecules via autophagy, a metabolic adaptation to nutrient deprivation, can also lead to immunotherapy resistance in pancreatic cancer (80). Inhibition of autophagy can restore cell surface levels of MHC class I, thereby enhancing antigen presentation and associated CD8^+^ T cell responses. Furthermore, in combination with dual immune checkpoint blockade therapy (anti-PD-1 and anti-CTLA-4 antibodies), treatment with an autophagy inhibitor, chloroquine, presents a synergistic effect in reducing tumor burden.

Microbiota as producers of immune-modulatory metabolites in the gut or in the TIME can directly affect tumor immunogenicity and thus efficacy of immunotherapy (81, 82). One prominent example is phytosphingosine, derived from *Lacticaseibacillus paracasei*, which activates the MyD88-NF-κB pathway to increase MHC class I expression and boosts ICI responses in breast cancer and melanoma (83). This study provides the underlying mechanisms of the clinically reported positive association between the enrichment of specific microbiota strains and responses to ICI-based therapy. As fecal microbiota transplantation (FMT) in ICI non-responders has shown clinical benefits in melanoma (84, 85), such mechanistic characterization will provide novel therapeutic combinations beyond FMT for ICI non-responders.

## Crosstalk between metabolite availability and epigenetic plasticity affects responses to immunotherapy

The interplay between metabolic adaptation and epigenetic reprogramming serves as one of the key regulatory mechanisms of cancer cell plasticity, including therapy resistance (14). Metabolic intermediates provide building blocks for writing epigenetic marks and cofactors for enzymes that erase epigenetic modifications. Notable examples include acyl-CoA metabolites that serve as donors for acylation (e.g. acetylation, lactylation, etc) and *S*-adenosylmethionine (SAM), which serves as a methyl door for histone, DNA, and RNA methylation, regulating gene transcription, translation, and RNA stability (86). On the other hand, epigenetic regulators modulate metabolic rewiring, enabling cancer cell survival and growth and promoting resistance to therapy (87).

In highly glycolytic liver cancer cells, expression of immunomodulatory molecule B7-H3 (CD276) is increased, suppressing anti-tumor immunity upon PD-1 blockade (88). Lactate, produced from glycolysis, promotes H3K18 lactylation at the *B7-H3* promoter, thereby upregulating B7-H3 expression and promoting evasion of CD8^+^ T cell-mediated cytotoxicity. Consistent with this mechanism, treatment with the LDH inhibitor, sodium oxamate in combination with anti-PD-1 therapy presents a synergistic effect in decreasing tumor burden *in vivo*.

Methionine serves as one of the key metabolic drivers in influencing methylation of histones and DNA, and thus epigenetically regulating gene transcription. Tumoral uptake of methionine through SLC43A2 deprives CD8^+^ T cells of methionine within the TIME, impairing cytotoxic T cell function (89). Methionine deficiency in CD8^+^ T cells decreases H3K79me2 levels and STAT5 transcription, resulting in T cell death and dysfunction. Pharmacological inhibition of tumor’s methionine importer by BCH (2-amino-2-norbornanecarboxylic acid) in combination with anti-PD-L1 presented a synergistic anti-tumor immune response *in vivo*.

Conversely, alterations in epigenetic regulators can reshape tumor metabolism and thereby affect responses to immunotherapy. One example is lysine demethylase 6A (KDM6A), an H3K27me2/me3 demethylase that is frequently mutated in bladder cancer (90). Loss of KDM6A in bladder cancer cells induces metabolic reprogramming characterized by reduced glycolysis and lactate production. The resulting decrease in lactate levels in TIME reduces H3K9 and H3K18 lactylation in regulatory T cells (Tregs), accompanied by decreased expression of Treg-associated immunosuppressive genes. As a result, loss of KDM6A enhances the response to anti-PD-1 therapy.

Epigenetic regulatory mechanisms underlying cellular defense against ferroptosis have been extensively studied, mainly focusing on regulation of cellular iron pools and the SLC7A11-glutathione-GPX4 system (91). Noncoding RNAs, including lncRNAs, can also regulate the ferroptosis sensitivity of cancer cells and thereby affect responses to immunotherapy, primarily by modulating components of the SLC7A11-glutathione-GPX4 defense system (92). For example, lncRNA SSTR5-AS1 enhances SLC7A11 transcription through STAT3 recruitment in ovarian cancer cell lines, resulting in resistance to ferroptosis and immune escape *in vitro* (93).

## Discussion

Utilizing tumor-intrinsic plasticity as a therapeutic vulnerability is a growing area of interest in tackling immunotherapy resistance. In this review, we highlight tumor-intrinsic plasticity, particularly epigenetic and metabolic reprogramming, as a key driver of immunotherapy resistance. Targeting these adaptive programs may enhance tumor sensitivity to existing immunotherapies and represents a promising strategy for overcoming resistance to immunotherapy. Currently, dozens of phase I and II clinical trials are evaluating combinations of immunotherapy with epidrugs [reviewed by Zhang et al. and Xu et al. (94, 95)] or metabolic modulators [reviewed by Yang et al. and Trefny et al. (96, 97)]. However, despite encouraging preclinical evidence, no such combination has yet been approved by the U.S. Food and Drug Administration (FDA). A major challenge in the clinical translation of these combination therapy approaches is the limited specificity of epidrugs and metabolic inhibitors. Since these agents can non-specifically target different cell types within the TIME, they may induce unintended molecular reprogramming in non-malignant cells, thereby compromising efficacy and increasing toxicity. Improving the targeted delivery of epidrugs or metabolic inhibitors can provide alternative routes to avoid these adverse effects. For example, antibody-drug conjugates can offer tumor cell-targeted delivery of epidrugs or metabolic inhibitors to overcome tumor-intrinsic immunotherapy resistance more effectively. Alternatively, epigenome editing technologies offer the potential to precisely modify epigenetic states in a tumor-specific manner.

Due to tumor-intrinsic plasticity, such combinatorial therapeutic strategies may also potentially lead to the development of acquired resistance. To better cope with or predict resistance to the combination treatment, utilization of emerging technologies such as single-cell RNA-sequencing, spatial transcriptomics, and spatial metabolomics will facilitate our understanding of how tumor-intrinsic adaptations reshape the TIME (98). In particular, these technologies may help resolve the spatial heterogeneity of tumor-intrinsic epigenetic and metabolic states and their interactions with immune cells within the TIME. Furthermore, machine learning and artificial intelligence (AI)-assisted platforms can link the complicated spatial patterns with clinical outcomes, promoting more rational clinical decision-making in immunotherapy treatment.

Molecular characterization of how epigenetic and metabolic reprogramming can regulate the response to immunotherapy in conjunction with *in vivo* validation of such combinatorial treatment options will open more therapeutic opportunities for patients refractory to immunotherapy. Technological advances in targeted drug delivery, precise modulation of epigenetic and metabolic programs, and accurate prediction of therapy outcome will benefit more cancer patients by enhancing tumor susceptibility to immunotherapy.

## References

[1] Leach DR Krummel MF Allison JP . Enhancement of antitumor immunity by CTLA-4 blockade. Science. (1996) 271:1734–6. doi: 10.1126/science.271.5256.1734 8596936

[2] Garon EB Rizvi NA Hui R Leighl N Balmanoukian AS Eder JP . Pembrolizumab for the treatment of non-small-cell lung cancer. N Engl J Med. (2015) 372:2018–28. doi: 10.1056/nejmoa1501824 25891174

[3] Hodi FS O'Day SJ McDermott DF Weber RW Sosman JA Haanen JB . Improved survival with ipilimumab in patients with metastatic melanoma. N Engl J Med. (2010) 363:711–23. doi: 10.1056/nejmoa1003466 20525992 PMC3549297

[4] Topalian SL Hodi FS Brahmer JR Gettinger SN Smith DC McDermott DF . Safety, activity, and immune correlates of anti-PD-1 antibody in cancer. N Engl J Med. (2012) 366:2443–54. doi: 10.1056/nejmoa1200690 22658127 PMC3544539

[5] Brahmer JR Tykodi SS Chow LQ Hwu WJ Topalian SL Hwu P . Safety and activity of anti-PD-L1 antibody in patients with advanced cancer. N Engl J Med. (2012) 366:2455–65. doi: 10.1056/nejmoa1200694 22658128 PMC3563263

[6] Ishida Y Agata Y Shibahara K Honjo T . Induced expression of PD-1, a novel member of the immunoglobulin gene superfamily, upon programmed cell death. EMBO J. (1992) 11:3887–95. doi: 10.1002/j.1460-2075.1992.tb05481.x 1396582 PMC556898

[7] Keir ME Butte MJ Freeman GJ Sharpe AH . PD-1 and its ligands in tolerance and immunity. Annu Rev Immunol. (2008) 26:677–704. doi: 10.1146/annurev.immunol.26.021607.090331 18173375 PMC10637733

[8] June CH Sadelain M . Chimeric antigen receptor therapy. N Engl J Med. (2018) 379:64–73. doi: 10.1056/nejmra1706169 29972754 PMC7433347

[9] Porter DL Levine BL Kalos M Bagg A June CH . Chimeric antigen receptor-modified T cells in chronic lymphoid leukemia. N Engl J Med. (2011) 365:725–33. doi: 10.1056/nejmoa1103849 21830940 PMC3387277

[10] Maude SL Frey N Shaw PA Aplenc R Barrett DM Bunin NJ . Chimeric antigen receptor T cells for sustained remissions in leukemia. N Engl J Med. (2014) 371:1507–17. doi: 10.1056/nejmoa1407222 25317870 PMC4267531

[11] Sharma P Hu-Lieskovan S Wargo JA Ribas A . Primary, adaptive, and acquired resistance to cancer immunotherapy. Cell. (2017) 168:707–23. doi: 10.1016/j.cell.2017.01.017 28187290 PMC5391692

[12] Chen DS Mellman I . Elements of cancer immunity and the cancer-immune set point. Nature. (2017) 541:321–30. doi: 10.1038/nature21349 28102259

[13] Kalbasi A Ribas A . Tumour-intrinsic resistance to immune checkpoint blockade. Nat Rev Immunol. (2020) 20:25–39. doi: 10.1038/s41577-019-0218-4 31570880 PMC8499690

[14] Choi J-E Baek I . Interplay between epigenetics and metabolism controls cancer stem cell plasticity. Front Epigenet Epigenomics. (2024) 2. doi: 10.3389/freae.2024.1424163 PMC13639278 42840520

[15] Chiappinelli KB Strissel PL Desrichard A Li H Henke C Akman B . Inhibiting DNA methylation causes an interferon response in cancer via dsRNA including endogenous retroviruses. Cell. (2015) 162:974–86. doi: 10.1016/j.cell.2017.03.036 26317466 PMC4556003

[16] Sheng W Lafleur MW Nguyen TH Chen S Chakravarthy A Conway JR . LSD1 ablation stimulates anti-tumor immunity and enables checkpoint blockade. Cell. (2018) 174:549–63:e19. doi: 10.1016/j.cell.2018.05.052 29937226 PMC6063761

[17] Burr ML Sparbier CE Chan KL Chan YC Kersbergen A Lam EYN . An evolutionarily conserved function of Polycomb silences the MHC class I antigen presentation pathway and enables immune evasion in cancer. Cancer Cell. (2019) 36:385–401:e8. doi: 10.1016/j.ccell.2019.08.008 31564637 PMC6876280

[18] Jones PA Ohtani H Chakravarthy A de Carvalho DD . Epigenetic therapy in immune-oncology. Nat Rev Cancer. (2019) 19:151–61. doi: 10.1038/s41568-019-0109-9 30723290

[19] Reinfeld BI Madden MZ Wolf MM Chytil A Bader JE Patterson AR . Cell-programmed nutrient partitioning in the tumour microenvironment. Nature. (2021) 593:282–8. doi: 10.1038/s41586-021-03442-1 33828302 PMC8122068

[20] Bader JE Voss K Rathmell JC . Targeting metabolism to improve the tumor microenvironment for cancer immunotherapy. Mol Cell. (2020) 78:1019–33. doi: 10.1016/j.molcel.2020.05.034 32559423 PMC7339967

[21] Hanahan D . Hallmarks of cancer-then and now, and beyond. Cell. (2026) 189:2254–77. doi: 10.1016/j.cell.2025.12.049 41616779

[22] Binnewies M Roberts EW Kersten K Chan V Fearon DF Merad M . Understanding the tumor immune microenvironment (TIME) for effective therapy. Nat Med. (2018) 24:541–50. doi: 10.1038/s41591-018-0014-x 29686425 PMC5998822

[23] Flavahan WA Gaskell E Bernstein BE . Epigenetic plasticity and the hallmarks of cancer. Science. (2017) 357:eaal2380. doi: 10.1126/science.aal2380 28729483 PMC5940341

[24] Topper MJ Vaz M Marrone KA Brahmer JR Baylin SB . The emerging role of epigenetic therapeutics in immuno-oncology. Nat Rev Clin Oncol. (2020) 17:75–90. doi: 10.1038/s41571-019-0266-5 31548600 PMC7254932

[25] Pan D Kobayashi A Jiang P Ferrari De Andrade L Tay RE Luoma AM . A major chromatin regulator determines resistance of tumor cells to T cell-mediated killing. Science. (2018) 359:770–5. doi: 10.1126/science.aao1710 29301958 PMC5953516

[26] Patel SJ Sanjana NE Kishton RJ Eidizadeh A Vodnala SK Cam M . Identification of essential genes for cancer immunotherapy. Nature. (2017) 548:537–42. doi: 10.1038/nature23477 28783722 PMC5870757

[27] Manguso RT Pope HW Zimmer MD Brown FD Yates KB Miller BC . *In vivo* CRISPR screening identifies Ptpn2 as a cancer immunotherapy target. Nature. (2017) 547:413–8. doi: 10.1038/nature23270 28723893 PMC5924693

[28] Jelinic P Ricca J van Oudenhove E Olvera N Merghoub T Levine DA . Immune-active microenvironment in small cell carcinoma of the ovary, hypercalcemic type: rationale for immune checkpoint blockade. J Natl Cancer Inst. (2018) 110:787–90. doi: 10.1093/jnci/djx277 29365144 PMC6037122

[29] Brodeur MN Dopeso H Zhu Y Longhini ALF Gazzo A Sun S . Interferon response and epigenetic modulation by SMARCA4 mutations drive ovarian tumor immunogenicity. Sci Adv. (2024) 10:eadk4851. doi: 10.1101/2023.08.08.552544 39630912 PMC11616711

[30] Sun D Qian H Wang J Xie T Teng F Li J . ARID1A deficiency reverses the response to anti-PD(L)1 therapy in EGFR-mutant lung adenocarcinoma by enhancing autophagy-inhibited type I interferon production. Cell Commun Signal. (2022) 20:156. doi: 10.1186/s12964-022-00958-5 36229854 PMC9558404

[31] Griffin GK Wu J Iracheta-Vellve A Patti JC Hsu J Davis T . Epigenetic silencing by SETDB1 suppresses tumour intrinsic immunogenicity. Nature. (2021) 595:309–14. doi: 10.1038/s41586-021-03520-4 33953401 PMC9166167

[32] Zhang SM Cai WL Liu X Thakral D Luo J Chan LH . KDM5B promotes immune evasion by recruiting SETDB1 to silence retroelements. Nature. (2021) 598:682–7. doi: 10.1038/s41586-021-03994-2 34671158 PMC8555464

[33] Wang D Quiros J Mahuron K Pai CC Ranzani V Young A . Targeting EZH2 reprograms intratumoral regulatory T cells to enhance cancer immunity. Cell Rep. (2018) 23:3262–74. doi: 10.1016/j.celrep.2018.05.050 29898397 PMC6094952

[34] Liu K Iyer R Li Y Zhu J Cai Z Wei J . USP22 drives tumor immune evasion and checkpoint blockade resistance through EZH2-mediated epigenetic silencing of MHC-I. J Clin Invest. (2026) 136(1):e193162. doi: 10.1172/jci193162 41252204 PMC12721901

[35] Cheng J He Z Jing J Liu Y Zhang H . Integrating machine learning and multi-omics to identify key SUMOylation molecular signature in sarcoma. Life Conflux. (2024) 1:e88. doi: 10.71321/lcflx.00001

[36] Demel UM Boger M Yousefian S Grunert C Zhang L Hotz PW . Activated SUMOylation restricts MHC class I antigen presentation to confer immune evasion in cancer. J Clin Invest. (2022) 132(9):e152383. doi: 10.1172/jci152383 35499080 PMC9057585

[37] Wang P Qiu J Fang Y Li S Liu K Cao Y . SENP3 inhibition suppresses hepatocellular carcinoma progression and improves the efficacy of anti-PD-1 immunotherapy. Cell Death Differ. (2025) 32:959–72. doi: 10.1038/s41418-024-01437-9 39755756 PMC12089275

[38] Mao Y Xia W Jiang P . Metabolites as signalling molecules in the tumour immune microenvironment. Nat Rev Immunol. (2026) 26:422–38. doi: 10.1038/s41577-025-01258-y 41526478

[39] Zhu W Fan C Hou Y Zhang Y . Lactylation in tumor microenvironment and immunotherapy resistance: New mechanisms and challenges. Cancer Lett. (2025) 627:217835. doi: 10.1016/j.canlet.2025.217835 40447130

[40] Zhu J Dai X Luo T Zhou Q Feng X Liu C . H3K18 lactylation drives CD8(+) T cell exclusion and anti-PD-1 resistance in hepatocellular carcinoma. Cell Rep. (2026) 45:117761. doi: 10.1016/j.celrep.2026.117761 42519827

[41] Liu S Pan Y Liu W Bu X Shao R Wang Q . Lactylation-driven MVP upregulation boosts immunotherapy resistance by inhibiting PD-L1 degradation in hepatocellular carcinoma. J Immunother Cancer. (2025) 13(9):e012230. doi: 10.1136/jitc-2025-012230 40983342 PMC12458742

[42] Xu YP Lv L Liu Y Smith MD Li WC Tan XM . Tumor suppressor TET2 promotes cancer immunity and immunotherapy efficacy. J Clin Invest. (2019) 129:4316–31. doi: 10.1172/jci129317 31310587 PMC6763236

[43] Huang C Gao Y Chen J Hong JH Jiang Y Chai KXY . Priming with DNMT inhibitors potentiates PD-1 immunotherapy by triggering viral mimicry in relapsed/refractory NK/T-cell lymphoma. Cancer Discov. (2025) 15:2450–67. doi: 10.1158/2159-8290.cd-25-0587 41088524 PMC12670078

[44] Topper MJ Vaz M Chiappinelli KB DeStefano Shields CE Niknafs N Yen RC . Epigenetic therapy ties MYC depletion to reversing immune evasion and treating lung cancer. Cell. (2017) 171:1284–300:e21. doi: 10.1016/j.cell.2017.10.022 29195073 PMC5808406

[45] Knox T Sahakian E Banik D Hadley M Palmer E Noonepalle S . Selective HDAC6 inhibitors improve anti-PD-1 immune checkpoint blockade therapy by decreasing the anti-inflammatory phenotype of macrophages and down-regulation of immunosuppressive proteins in tumor cells. Sci Rep. (2019) 9:6136. doi: 10.1038/s41598-019-42237-3 30992475 PMC6467894

[46] Fang Z Li J Xiong Y Luo P Lu Q Wu M . FBXL16 regulates TAMs recruitment by mediating cytokine release in gliomas. Cancer Genet. (2025) 298-299:285–94. doi: 10.1016/j.cancergen.2025.11.006 41265012

[47] Zhou L Mudianto T Ma X Riley R Uppaluri R . Targeting EZH2 enhances antigen presentation, antitumor immunity, and circumvents anti-PD-1 resistance in head and neck cancer. Clin Cancer Res. (2020) 26:290–300. doi: 10.1158/1078-0432.ccr-19-1351 31562203 PMC6942613

[48] Qiu F Yang Q Sun W Ruan K Jiang N Zhou J . EZH2 inhibition activates dsRNA-interferon axis stress and promotes response to PD-1 checkpoint blockade in NSCLC. J Cancer. (2022) 13:2893–904. doi: 10.7150/jca.73291 35912007 PMC9330466

[49] Ducote TJ Song X Naughton KJ Chen F Plaugher DR Childress AR . EZH2 inhibition promotes tumor immunogenicity in lung squamous cell carcinomas. Cancer Res Commun. (2024) 4:388–403. doi: 10.1158/2767-9764.crc-23-0399 38265267 PMC10863487

[50] Spiliopoulou P Spear S Mirza H Garner I McGarry L Grundland-Freile F . Dual G9A/EZH2 inhibition stimulates antitumor immune response in ovarian high-grade serous carcinoma. Mol Cancer Ther. (2022) 21:522–34. doi: 10.1158/1535-7163.mct-21-0743 35131874 PMC9377747

[51] Chang CH Qiu J O'Sullivan D Buck MD Noguchi T Curtis JD . Metabolic competition in the tumor microenvironment is a driver of cancer progression. Cell. (2015) 162:1229–41. doi: 10.1016/j.cell.2015.08.016 26321679 PMC4864363

[52] Cascone T McKenzie JA Mbofung RM Punt S Wang Z Xu C . Increased tumor glycolysis characterizes immune resistance to adoptive T cell therapy. Cell Metab. (2018) 27:977–87:e4. doi: 10.1016/j.cmet.2018.02.024 29628419 PMC5932208

[53] Brand A Singer K Koehl GE Kolitzus M Schoenhammer G Thiel A . LDHA-associated lactic acid production blunts tumor immunosurveillance by T and NK cells. Cell Metab. (2016) 24:657–71. doi: 10.1016/j.cmet.2016.08.011 27641098

[54] Pilon-Thomas S Kodumudi KN El-Kenawi AE Russell S Weber AM Luddy K . Neutralization of tumor acidity improves antitumor responses to immunotherapy. Cancer Res. (2016) 76:1381–90. doi: 10.1158/0008-5472.can-15-1743 26719539 PMC4829106

[55] Babl N Decking SM Voll F Althammer M Sala-Hojman A Ferretti R . MCT4 blockade increases the efficacy of immune checkpoint blockade. J Immunother Cancer. (2023) 11(10):e007349. doi: 10.1136/jitc-2023-007349 37880183 PMC10603342

[56] Cappellesso F Orban MP Shirgaonkar N Berardi E Serneels J Neveu MA . Targeting the bicarbonate transporter SLC4A4 overcomes immunosuppression and immunotherapy resistance in pancreatic cancer. Nat Cancer. (2022) 3:1464–83. doi: 10.1038/s43018-022-00470-2 36522548 PMC9767871

[57] Ding S Wang H Han Z Hong S . Immunomodulation by glycosylation: bridging glycobiology to adaptive immunity and therapeutic innovation. Front Immunol. (2026) 17:1729274. doi: 10.3389/fimmu.2026.1729274 41939861 PMC13047073

[58] Greco B Malacarne V De Girardi F Scotti GM Manfredi F Angelino E . Disrupting N-glycan expression on tumor cells boosts chimeric antigen receptor T cell efficacy against solid malignancies. Sci Transl Med. (2022) 14:eabg3072. doi: 10.1126/scitranslmed.abg3072 35044789

[59] Hollander EE Flock RE McDevitt JC Vostrejs WP Campbell SL Orlen MI . N-glycosylation by Mgat5 imposes a targetable constraint on immune-mediated tumor clearance. JCI Insight. (2024) 9(12):e178804. doi: 10.1172/jci.insight.178804 38912584 PMC11383181

[60] Ghasempour S Freeman SA . The glycocalyx and immune evasion in cancer. FEBS J. (2023) 290:55–65. doi: 10.1111/febs.16236 34665926

[61] Park S Ho CE Darnell EP Wolff AN Takei H Birocchi F . Tuning CAR-T cells by targeting cancer-associated glycan in pancreatic cancer. Nat Commun. (2025) 16:11246. doi: 10.1038/s41467-025-66102-2 41372740 PMC12717153

[62] Erler P Kurcon T Cho H Skinner J Dixon C Grudman S . Multi-armored allogeneic MUC1 CAR T cells enhance efficacy and safety in triple-negative breast cancer. Sci Adv. (2024) 10:eadn9857. doi: 10.1126/sciadv.adn9857 39213364 PMC11364110

[63] Posey AD Schwab RD Boesteanu AC Steentoft C Mandel U Engels B . Engineered CAR T cells targeting the cancer-associated Tn-glycoform of the membrane mucin MUC1 control adenocarcinoma. Immunity. (2016) 44:1444–54. doi: 10.1016/j.immuni.2016.05.014 27332733 PMC5358667

[64] Supimon K Sangsuwannukul T Sujjitjoon J Phanthaphol N Chieochansin T Poungvarin N . Anti-mucin 1 chimeric antigen receptor T cells for adoptive T cell therapy of cholangiocarcinoma. Sci Rep. (2021) 11:6276. doi: 10.1038/s41598-021-85747-9 33737613 PMC7973425

[65] Guo C You Z Shi H Sun Y Du X Palacios G . SLC38A2 and glutamine signalling in cDC1s dictate anti-tumour immunity. Nature. (2023) 620:200–8. doi: 10.1038/s41586-023-06299-8 37407815 PMC10396969

[66] Edwards DN Ngwa VM Raybuck AL Wang S Hwang Y Kim LC . Selective glutamine metabolism inhibition in tumor cells improves antitumor T lymphocyte activity in triple-negative breast cancer. J Clin Invest. (2021) 131(4):e140100. doi: 10.1172/jci140100 33320840 PMC7880417

[67] Leone RD Zhao L Englert JM Sun IM Oh MH Sun IH . Glutamine blockade induces divergent metabolic programs to overcome tumor immune evasion. Science. (2019) 366:1013–21. doi: 10.1126/science.aav2588 31699883 PMC7023461

[68] Dixon SJ Lemberg KM Lamprecht MR Skouta R Zaitsev EM Gleason CE . Ferroptosis: an iron-dependent form of nonapoptotic cell death. Cell. (2012) 149:1060–72. doi: 10.1016/j.cell.2012.03.042 22632970 PMC3367386

[69] Chen X Kang R Kroemer G Tang D . Broadening horizons: the role of ferroptosis in cancer. Nat Rev Clin Oncol. (2021) 18:280–96. doi: 10.1038/s41571-020-00462-0 33514910

[70] Wang W Green M Choi JE Gijón M Kennedy PD Johnson JK . CD8(+) T cells regulate tumour ferroptosis during cancer immunotherapy. Nature. (2019) 569:270–4. doi: 10.1038/s41586-019-1170-y 31043744 PMC6533917

[71] Cramer SL Saha A Liu J Tadi S Tiziani S Yan W . Systemic depletion of L-cyst(e)ine with cyst(e)inase increases reactive oxygen species and suppresses tumor growth. Nat Med. (2017) 23:120–7. doi: 10.1038/nm.4232 27869804 PMC5218918

[72] Chen S Hu T Zhu K Liu Y Yang Y Li X . 16-h fasting optimizes cancer immunotherapy in mice and humans. Cell Metab. (2026) 38:944–62:e14. doi: 10.1016/j.cmet.2026.01.015 41720105

[73] Xiang J Wang T Tian S Li J Luo M Wang Y . Aberrant amino acid-sensing promotes immunotherapy resistance via the inflammatory cytokine-ZBTB5-mTORC1 axis. Nat Cell Biol. (2026) 28:1000–14. doi: 10.1038/s41556-026-01926-8 41933211

[74] Bell HN Huber AK Singhal R Korimerla N Rebernick RJ Kumar R . Microenvironmental ammonia enhances T cell exhaustion in colorectal cancer. Cell Metab. (2023) 35:134–49:e6. doi: 10.1016/j.cmet.2022.11.013 36528023 PMC9841369

[75] Wei H Niu C Shi Y Fang Y Yang C Liu J . Mogat1 drives metabolic adaptations to evade immune surveillance. Nat Commun. (2025) 16:7035. doi: 10.1038/s41467-025-62134-w 40745160 PMC12314084

[76] Sade-Feldman M Jiao YJ Chen JH Rooney MS Barzily-Rokni M Eliane JP . Resistance to checkpoint blockade therapy through inactivation of antigen presentation. Nat Commun. (2017) 8:1136. doi: 10.1038/s41467-017-01062-w 29070816 PMC5656607

[77] Nielsen M Presti M Sztupinszki Z Jensen AWP Draghi A Chamberlain CA . Coexisting alterations of MHC Class I antigen presentation and IFNgamma signaling mediate acquired resistance of melanoma to post-PD-1 immunotherapy. Cancer Immunol Res. (2022) 10:1254–62. doi: 10.1158/2326-6066.cir-22-0326 35969233

[78] Gettinger S Choi J Hastings K Truini A Datar I Sowell R . Impaired HLA class I antigen processing and presentation as a mechanism of acquired resistance to immune checkpoint inhibitors in lung cancer. Cancer Discov. (2017) 7:1420–35. doi: 10.1158/2159-8290.cd-17-0593 29025772 PMC5718941

[79] Paulson KG Voillet V McAfee MS Hunter DS Wagener FD Perdicchio M . Acquired cancer resistance to combination immunotherapy from transcriptional loss of class I HLA. Nat Commun. (2018) 9:3868. doi: 10.1038/s41467-018-06300-3 30250229 PMC6155241

[80] Yamamoto K Venida A Yano J Biancur DE Kakiuchi M Gupta S . Autophagy promotes immune evasion of pancreatic cancer by degrading MHC-I. Nature. (2020) 581:100–5. doi: 10.1038/s41586-020-2229-5 32376951 PMC7296553

[81] Toner-Bartelds C Mimpen IL Parra-Martinez M Burgering BMT Voest EE . Microbiota-derived metabolites as modulators of cancer immunotherapy response. Nat Commun. (2026) 17:5274. doi: 10.1038/s41467-026-72178-1 42014741 PMC13269703

[82] Gazzaniga FS Kasper DL . The gut microbiome and cancer response to immune checkpoint inhibitors. J Clin Invest. (2025) 135(3):e184321. doi: 10.1172/jci184321 39895632 PMC11785914

[83] Ferrari V Lo Cascio A Melacarne A Tanasković N Mozzarelli AM Tiraboschi L . Sensitizing cancer cells to immune checkpoint inhibitors by microbiota-mediated upregulation of HLA class I. Cancer Cell. (2023) 41:1717–30:e4. doi: 10.1016/j.ccell.2023.08.014 37738976

[84] Baruch EN Youngster I Ben-Betzalel G Ortenberg R Lahat A Katz L . Fecal microbiota transplant promotes response in immunotherapy-refractory melanoma patients. Science. (2021) 371:602–9. doi: 10.1126/science.abb5920 33303685

[85] Davar D Dzutsev AK McCulloch JA Rodrigues RR Chauvin JM Morrison RM . Fecal microbiota transplant overcomes resistance to anti-PD-1 therapy in melanoma patients. Science. (2021) 371:595–602. doi: 10.1126/science.abf3363 33542131 PMC8097968

[86] Boon R Silveira GG Mostoslavsky R . Nuclear metabolism and the regulation of the epigenome. Nat Metab. (2020) 2:1190–203. doi: 10.1038/s42255-020-00285-4 33046909

[87] Izzo LT Affronti HC Wellen KE . The bidirectional relationship between cancer epigenetics and metabolism. Annu Rev Cancer Biol. (2021) 5:235–57. doi: 10.1146/annurev-cancerbio-070820-035832 34109280 PMC8186467

[88] Ma Z Yang J Jia W Li L Li Y Hu J . Histone lactylation-driven B7-H3 expression promotes tumor immune evasion. Theranostics. (2025) 15:2338–59. doi: 10.7150/thno.105947 39990209 PMC11840737

[89] Bian Y Li W Kremer DM Sajjakulnukit P Li S Crespo J . Cancer SLC43A2 alters T cell methionine metabolism and histone methylation. Nature. (2020) 585:277–82. doi: 10.1038/s41586-020-2682-1 32879489 PMC7486248

[90] Singh P D'Rozario R Chakraborty B Meher S Raychaudhuri D Tannir AJ . Loss of KDM6A-mediated genomic instability and metabolic reprogramming regulates response to therapeutic perturbations in bladder cancer. Nat Commun. (2026) 17:1382. doi: 10.1038/s41467-025-68132-2 41501085 PMC12877084

[91] Wang Y Hu J Wu S Fleishman JS Li Y Xu Y . Targeting epigenetic and posttranslational modifications regulating ferroptosis for the treatment of diseases. Signal Transduction Targeted Ther. (2023) 8:449. doi: 10.1038/s41392-023-01720-0 38072908 PMC10711040

[92] Balihodzic A Prinz F Dengler MA Calin GA Jost PJ Pichler M . Non-coding RNAs and ferroptosis: potential implications for cancer therapy. Cell Death Differ. (2022) 29:1094–106. doi: 10.1038/s41418-022-00998-x 35422492 PMC9177660

[93] Wei Q Yang Y Wang H Li C Li Y . The mechanism of lncRNA SSTR5-AS1 promoting ferroptosis resistance and immune escape in ovarian cancer cells by recruiting STAT3 to regulate SLC7A11 expression. Cancer Genet. (2025) 296-297:172–81. doi: 10.1016/j.cancergen.2025.07.009 40716132

[94] Zhang H Pang Y Yi L Wang X Wei P Wang H . Epigenetic regulators combined with tumour immunotherapy: Current status and perspectives. Clin Epigenet. (2025) 17:51. doi: 10.1186/s13148-025-01856-6 40119465 PMC11929245

[95] Xu Y Li P Liu Y Xin D Lei W Liang A . Epi-immunotherapy for cancers: rationales of epi-drugs in combination with immunotherapy and advances in clinical trials. Cancer Commun (Lond). (2022) 42:493–516. doi: 10.1002/cac2.12313 35642676 PMC9198339

[96] Yang L Zhang C Zhang Z Yao S Shen J Zhang Z . Targeting metabolic reprogramming to enhance adoptive immunotherapy: Emerging mechanisms and translational perspectives. J Transl Med. (2026) 24(1):476. doi: 10.1186/s12967-026-07887-8 41764546 PMC13059335

[97] Trefny MP Kroemer G Zitvogel L Kobold S . Metabolites as agents and targets for cancer immunotherapy. Nat Rev Drug Discov. (2025) 24:764–84. doi: 10.1038/s41573-025-01227-z 40571788

[98] Shi T Guo Y Tang X Liu Z Guo Q Fang T . Decoding tumor immune microenvironment heterogeneity by single-cell and spatial multi-omics: From immunotherapy resistance to translational biomarkers. Neoplasia. (2026) 80:101348. doi: 10.1016/j.neo.2026.101348 42594562 PMC13487894

